# Sporadic Creutzfeldt-Jakob Disease: Finding the Needle in the Haystack

**DOI:** 10.7759/cureus.64548

**Published:** 2024-07-15

**Authors:** Auns Ghazanfar, Alexandra Pittford, Kryshani Fernando

**Affiliations:** 1 Internal Medicine, University Hospital Sussex National Health Services (NHS) Foundation Trust, Chichester, GBR; 2 Stroke Medicine, University Hospital Sussex National Health Services (NHS) Foundation Trust, Chichester, GBR; 3 Neurology, University Hospital Sussex National Health Services (NHS) Foundation Trust, Chichester, GBR

**Keywords:** neuro-psychiatric, gait disturbance, personality disrders, memory issues, creutzfeld-jakob disease

## Abstract

Sporadic Creutzfeldt-Jakob disease (SCJD) is a rare neurodegenerative disease with a very low prevalence. The aetiology is theorised to be genetic. Modern laboratory techniques, such as the real-time quaking-induced conversion (RT-QuIC) assay, have allowed us to diagnose CJD with greater sensitivity and specificity. Previously, the diagnosis rested primarily on a post-mortem brain biopsy. Although advancements in laboratory techniques have allowed earlier diagnosis of CJD, the treatment is still supportive. Research is still ongoing for a curative treatment, but so far, the fatality rate remains at 100%. Early vague symptoms of CJD delay the diagnosis further, as multiple pathologies need to be ruled out before consideration of the diagnosis of CJD. This case report describes a similar case of sporadic CJD diagnosed in an otherwise fit and well patient.

## Introduction

Creutzfeldt-Jakob disease (CJD) is a fatal neurodegenerative disorder that initially presents with a variety of non-specific symptoms, such as loss of intellect and memory, with subtle personality changes, making it very challenging to diagnose in non-specialist centres. Recent advancements in laboratory testing techniques, such as real-time quaking-induced conversion (RT-QuIC) assays, have shown promising results, with high sensitivity and specificity for cerebrospinal fluid (CSF) samples of patients with CJD. The gold standard for the diagnosis of CJD remains a brain biopsy. The prevalence of CJD is one to two cases per million people every year. In the U.K., the National CJD Research and Surveillance Unit (NCJDRSU) actively monitors all new and diagnosed cases. In 2023, there were 153 cases of suspected CJD and 132 cases of definite or probable CJD in the UK [1]. This correlates with the rare prevalence of the disease in other countries. We present a case of sporadic CJD that was admitted to our local general hospital.

## Case presentation

A 57-year-old male was initially brought to his general practitioner (GP) by his wife in May 2022 for problems with his memory for the past six months. He first noted he had short-term memory loss in November 2021, where he would forget things, he had said or done. He then developed difficulty sleeping and would be up wandering around the house at night. His memory issues worsened over the months. At the time, he had some stress from his job, so he was prescribed mirtazapine by his general practitioner (GP). However, along with his memory issues, he developed personality changes where he would become irritable, frustrated, and aggressive over small things. He had a heated argument with his son in late April 2022, in which he nearly became physical, and his wife moved out. Since then, he started having delusions and would often confabulate. After May 2022, he also developed cerebellar symptoms, had an ataxic, slow-shuffling gait, and would often fall. He was initially admitted to the hospital on August 9, 2022, with confusion. He was reviewed by neurology and then discharged to do an MRI of the brain as an outpatient. However, he was re-admitted on August 26, 2022, for increasing confusion and paranoia.

The patient had a background of ischaemic heart disease, hypertension, depression, gout, type 2 diabetes, and fatty liver disease. He previously drank 15-20 pints of beer a week. He has previously had nasal cautery but had no major operations and has no risk factors for iatrogenic or variant CJD. He had three children who were in their 20s. He had one sister who was 62 and had no history of dementia or neurological diagnoses. His mother passed away in her late 70s with a diagnosis of vascular dementia, which progressed over 10 years. His father was aged 85 and received a diagnosis of non-specified dementia about three years prior.

On examination, he was restless and fidgety. His speech was slurred. He had normal eye movements and normal power, tone, and reflexes. There was no myoclonus or primitive reflexes. He was impaired on dysdiadochokinesis, had an intention tremor, and was unable to perform the Luria test. He was observed walking a few steps with a Zimmer frame; his gait was broad-based and shuffling. He scored 17/20 on the MRC cognitive scale, dropping marks on verbal recognition memory (9/12), fatigue assessment scale (FAS) testing (14't' words in 1 minute), and visually fragmented letters (B/9).

During admission, he required a Zimmer frame to mobilize; he had been restless and agitated at times in the hospital and had occasionally required lorazepam for this. He had two episodes where he became less responsive and had a left-sided weakness, recovering slowly over a few hours; all investigations done at the time of these episodes were normal. His delusions became worse with disinhibition, and he developed expressive language problems.

**Figure 1:**
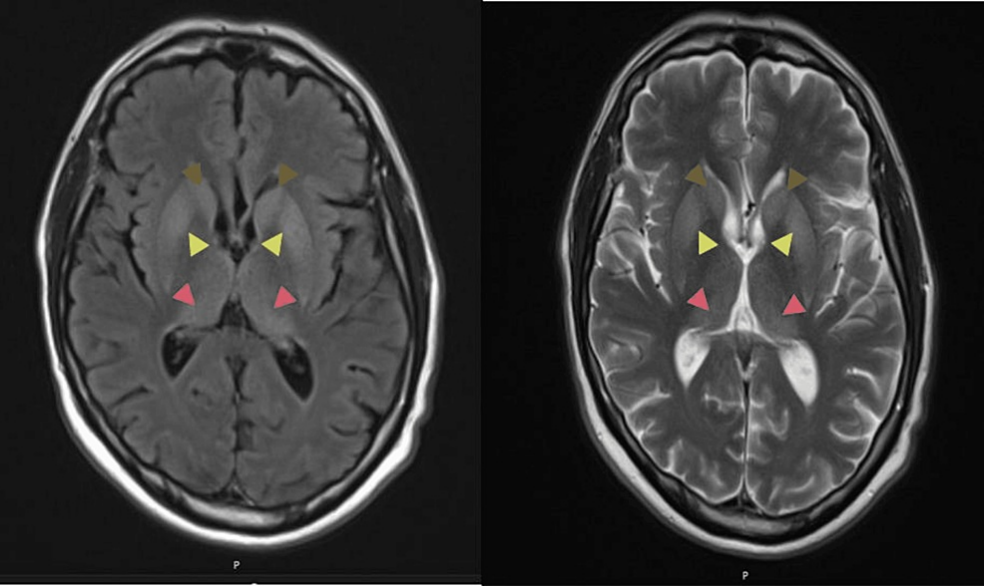
MRI images of the patient Pathological T2/FLAIR high signal on MRI within the deep grey matter, in particular the thalamus. Hyperdensities can be seen in the caudate nucleus bilaterally (marked by brown arrows), in the putamen nucleus of the thalamus bilaterally (marked by yellow arrows), and bilaterally in the pulvinar thalamic nuclei (marked by red arrows). The bilateral hyperdensities of the pulvinar nuclei are classically known as the 'pulvinar sign'.

He had magnetic resonance imaging (MRI) of his head on August 18, 2022, which showed a high pathological T2/FLAIR (fluid-attenuated inversion recovery) signal within the deep grey matter, in particular the caudate nucleus, striatum, and thalamus on both sides, associated with further signal abnormality within the parahippocampal gyrus bilaterally. These findings were suspicious of a possible CJD. The MRI images are shown below in Figure 1. 

This prompted the hospital team to refer the patient to the National CJD Research and Surveillance Unit (NCJDRSU). He then had the following investigations summarised in Table 1.

**Table 1:** Blood and cerebrospinal (CSF) investigations VZV: Varicella zoster virus, HSV: Herpes simplex virus, VGKC: Voltage-gated potassium channel, LGI1: Leucine-rich glioma-inactivated 1, CASPR2: Contactin-associated protein-2, NMDA: N-methyl-D-aspartate Antibodies, TSH: Thyroid stimulating hormone

Blood workup	Cereberospinal fluid workup (CSF)
Ammonia 31 ꭒmol/L	Protein 0.67 g/L
Copper 16.3 ꭒmol/L	White blood cells 1.0 mmᶟ
Hepatitis Screen negative	Red blood cells 162.0 mmᶟ
Vitamin B12 300 ng/L and Folate 12.1 ꭒmol/L	Glucose 4.9 mmol/L
TSH 0.62 mu/L and free T3 4.3 pmol/L	CSF virology: VZV, HSV, Enterovirus not detected
Caeruloplasmin 0.28 g/L	CSF culture: No growth after 2 days
Serology of HIV and Syphilis Negative	
Serum VGKC, LGI1, CASPR2, NMDA Antibodies- Negative	
Paraneoplastic Antibodies Hu, Ri, Yo - Negative	
Autoimmune screen- CCP Abs, PR3 Abs, MPO Abs- Negative	
Rheumatoid factor Negative	
Connective tissues disease screen negative	
Imunoglubulins-IgA,IgM and IgG-normal	
Creatinine Kinase 191 U/L	

His CT thorax abdomen pelvis with contrast showed no sinister lesion, and electroencephalography (EEG) showed non-specific findings with no diagnostic epileptiform or focal abnormality.

Based on the clinical findings and the investigations, an RT-QuIC CSF sample was sent to Edinburgh (NCJDRSU). NCJDRSU also advised getting PRNP gene testing to exclude any genetic variant of CJD from University College London Hospital (UCLH).

As there was no treatment for CJD, the patient was seen by the hospital palliative team to ensure he remained comfortable. He was eventually discharged to a care home for end-of-life care with anticipatory medications. His symptoms eventually got worse, and he passed away 11 days later.

This case was unique as sporadic CJD typically has a much more progressive symptom onset, and death occurs in four to six months, whereas in this case, a period of 18 months has passed since the onset of symptoms and death.

His RT-QuIC test came back positive in January 2023, confirming the diagnosis of sporadic CJD.

## Discussion

CJD, although a rare disease, has a fatality rate of 100%, with no curative treatment developed so far. CJD can be classified into three main types: sporadic CJD, which accounts for 85% of all cases; genetic or familial CJD, which accounts for about 10%-15% of the cases; and infectious (acquired) CJD, in which a prion acquires it from an external source, which accounts for less than 1% of all cases [2]. Acquired CJD can further be divided into variant CJD (vCJD) and iatrogenic CJD (iCJD). vCJD is transmitted by consumption of infected meat, such as cattle or beef; it is rarely seen now, as, after 1988, the UK government took strict measures to ensure that CJD is not transmitted to humans by infected animals [3]. iCJD cases have also dropped as advancements in technology have now allowed for the production of synthetic products rather than using cadaveric material in clinical practice.

**Figure 2:**
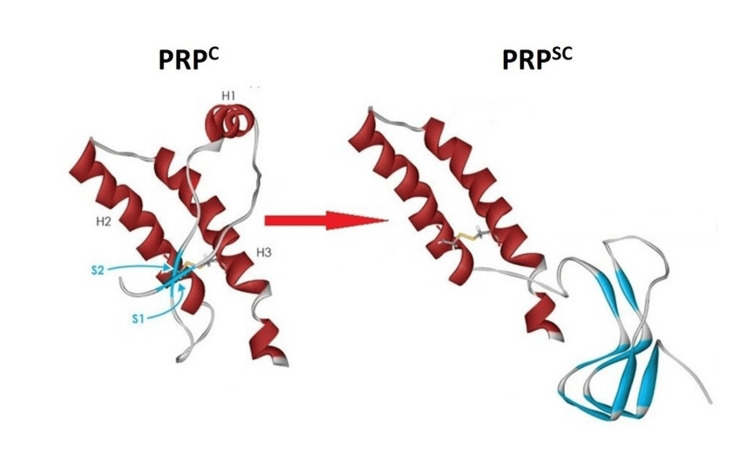
Conversion from PrPC to PrPSc Normal and infective forms normal prion protein (PrPC) with 43% of α-helix, sensitive to proteinase K treatment without forming aggregates, to (PrPSc) human prion protein infectious isoform with 30% of α-helix and 43% of β-sheet, resistant to proteinase K treatment and capable of forming aggregates. *(Picture taken from the article Prion/Virus: The Dangers of Biological Weapons.)  World Journal of Biology, Pharmacy, and Health Sciences (with permission from the author, Eric Almeida Xavier).*

CJD is primarily a prion neurodegenerative disease. A prion is a protein particle distinct from other infectious organisms. PrPC is a glycoprotein normally found in human neuron cells with a predominant A-helical structure, which is vital for neuronal signal transmission and prevents the apoptotic death of neurons. It is majorly hydrophilic in structure and is coded by the PRNP gene on chromosome 20 in humans [4]. A prion causes the predominated A-helical structure of PrPC to be converted into pathological PrPSc (Scrapie isoform), which mostly has a B-pleated sheet structure. PrPSc is different from PrPC in terms of its solubility and its resistance to protease [5]. The PrPSc scrapie glycoprotein then self-propagates and infects other PrPC, converting them into the PrPSc scrapie isoform. Rapid loss of neurons then gives a spongiform appearance to the brain on histopathology [6]. This conversion from PrPC to PrPSc and its structure is summarised in Figure 2 (image courtesy of Eric Xavier from her publication Prion/Virus: The Dangers of Biological Weapons). (World Journal of Biology, Pharmacy, and Health Sciences).

In the sporadic form of CJD, no apparent cause or trigger is identified for transmission of prion. Sporadic CJD can further be classified into two types based on codon 129 on the PRNP gene, which can either code for methionine or valine amino acids and two different types of protease-resistant prion protein core [7].

The diagnosis of CJD can be very challenging as it can present with a variety of vague symptoms initially, such as subtle personality changes, short-term memory loss, and sleep disturbances. The symptoms can mimic a variety of disorders, such as depression, infective/autoimmune encephalitis, Wilson’s disease, dementia, vasculitis, and paraneoplastic syndromes.

**Figure 3:**
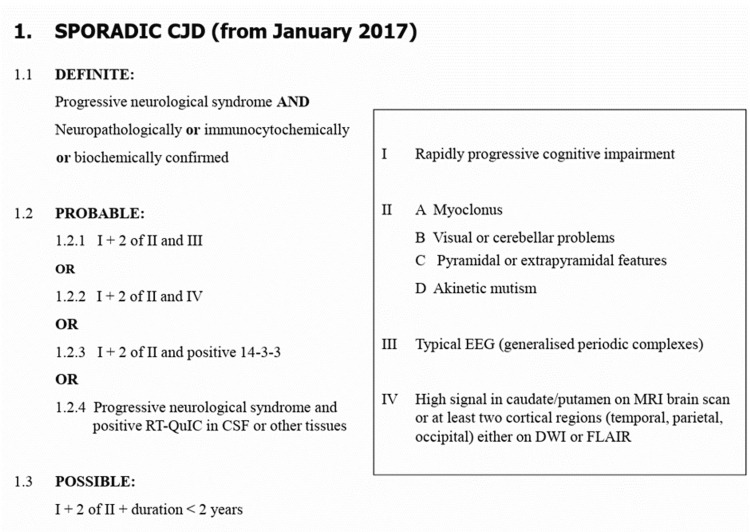
NCJDRSU criteria which classifying the cases as definite, probable, and possible Sporadic CJD Official Criteria for Diagnosis of Sporadic CJD from the NCJDRSU Public Website

Sporadic CJD is a notifiable disease. The NCJDRSU has set out a criterion that classifies cases as definite, probable, and possible sporadic CJD. The criteria are shown in Figure 3 (image taken from the NCJDRSU website):

To reach a definite diagnosis of antemortem, we must rule out all other causes of symptoms, which requires extensive investigations. MRI heads (T2, FLAIR, and DWI) in sporadic CJD typically show bilateral, symmetric, and hyperintense signal changes in the basal ganglia and cortical regions [8]. The most common parts affected are the insula, cingulate gyrus, superior frontal gyrus, caudate, putamen, and thalamus [9].

As a result of damaged neuronal cells, CSF 14-3-3 is raised; however, recent promising techniques such as RT-QuIC are much more sensitive (94%) and specific (100%) in diagnosing sporadic CJD [10].

However, with recent advances in diagnosing sporadic CJD, the fatality remains 100%, and to date, there is no curative treatment available. Patients are treated with supportive management and a palliative approach. There are ongoing clinical trials on the use of a humanised anti-PrPC monoclonal antibody (an IgG4 κ isotype; PRN100), which has shown some efficacy against sCJD, but it is still a long way for a definite treatment guideline to be released [11].

## Conclusions

The case described above signifies that although rare pathology, CJD should be considered in a list of diagnoses in patients with cognitive impairment who are otherwise fit and healthy. It should be emphasised that clinical practice and theoretical knowledge can sometimes vary from case to case. In this case, the disease progressed over 18 months, whereas classically, death from sCJD occurs within four to six months. Early detection is important; therefore, an early discussion with specialist centres regarding relevant investigations is paramount. A multi-disciplinary and holistic approach is required in such cases with no curative treatment, as patients are usually young with dependents who will require support after such an untimely and unexpected diagnosis. The lack of curative treatment emphasises the role of research to ensure new therapies and guidelines can be made that will not only prevent mortality but also improve quality of life.
